# Giant intrathoracic benign tumors: more threat than a challenge

**DOI:** 10.1186/1749-8090-8-S1-O233

**Published:** 2013-09-11

**Authors:** C Tunea, O Burlacu, V Voiculescu, G Cozma, I Miron, I Petrache, A Nicodin

**Affiliations:** 1 Department of Thoracic Surgery, Emergency Municipal Hospital, Timisoara, Romania; 2 University of Medicine, Timisoara, Romania

## Background

Giant benign intra-thoracic tumors are very rare. The symptoms are non-specific, the imagistic is impressive, though the surgical ablation and the postoperative outcome are simple.

## Methods

We reviewed retrospectively the charts of 16 patients, admitted between 2001 and 2012, aged 16 and 68 years. The diagnostic was based on imagistic study. We used open surgery from the beginning. We analyzed average with standard deviation for age, postoperative in hospital stay, correlation between the size and hospital stay, age and hospital stay, age and tumor size.

## Results

The mean age was 42,6 ± 16,4 years, with male/female ratio of 0.77. The mean postoperative hospital stay was 8,5 ± 2,52 days and the correlation between tumor size and hospital stay was 0.005. The mean size of the tumors was 13.31 ± 5,4 cm. Correlation between age and tumor size 3.319 and between age and hospital stay 0.032. We performed complete resection via postero-lateral approach with active suction. With the exception of one case with slow lung re-expansion (tumor over 20 cm) we recorded no postoperative morbidity.

## Conclusions

Although the preoperative imagistic aspects of this pathology are very impressive, the technique represents virtually no challenge for the surgeon.

